# Skeleton Driven Action Recognition Using an Image-Based Spatial-Temporal Representation and Convolution Neural Network

**DOI:** 10.3390/s21134342

**Published:** 2021-06-25

**Authors:** Vinícius Silva, Filomena Soares, Celina P. Leão, João Sena Esteves, Gianni Vercelli

**Affiliations:** 1 Centro Algoritmi, University of Minho, Campus of Azurém, 4800-058 Guimarães, Portugal; a65312@alunos.uminho.pt (V.S.); cpl@dps.uminho.pt (C.P.L.); sena@dei.uminho.pt (J.S.E.); 2 DIBRIS, University of Genoa, 13-16145 Genoa, Italy; gianni.vercelli@unige.it

**Keywords:** human action recognition, human computer interaction, autism spectrum disorder, convolutional neural network

## Abstract

Individuals with Autism Spectrum Disorder (ASD) typically present difficulties in engaging and interacting with their peers. Thus, researchers have been developing different technological solutions as support tools for children with ASD. Social robots, one example of these technological solutions, are often unaware of their game partners, preventing the automatic adaptation of their behavior to the user. Information that can be used to enrich this interaction and, consequently, adapt the system behavior is the recognition of different actions of the user by using RGB cameras or/and depth sensors. The present work proposes a method to automatically detect in real-time typical and stereotypical actions of children with ASD by using the Intel RealSense and the Nuitrack SDK to detect and extract the user joint coordinates. The pipeline starts by mapping the temporal and spatial joints dynamics onto a color image-based representation. Usually, the position of the joints in the final image is clustered into groups. In order to verify if the sequence of the joints in the final image representation can influence the model’s performance, two main experiments were conducted where in the first, the order of the grouped joints in the sequence was changed, and in the second, the joints were randomly ordered. In each experiment, statistical methods were used in the analysis. Based on the experiments conducted, it was found statistically significant differences concerning the joints sequence in the image, indicating that the order of the joints might impact the model’s performance. The final model, a Convolutional Neural Network (CNN), trained on the different actions (typical and stereotypical), was used to classify the different patterns of behavior, achieving a mean accuracy of 92.4% ± 0.0% on the test data. The entire pipeline ran on average at 31 FPS.

## 1. Introduction

Autism Spectrum Disorder (ASD) is a lifelong disability that affects people’s ability to communicate and to understand social cues. Consequently, individuals with ASD are unable to interact socially with their peers successfully. It affects 1 in 54 children and it is four times more common among boys than girls [1]. Furthermore, individuals with ASD demonstrate stereotypical behaviors that are defined as restricted and repetitive patterns that appear to the observer to be invariant in form and without any obvious eliciting stimulus [2]. Stereotypical actions are defined as being repetitive and sometimes inappropriate in nature [3]. They are highly heterogeneous in presentation, being verbal or non-verbal, fine or gross motor-oriented, as well as simple or complex. Frequent examples of stereotyped behaviors are hand flapping/wave, body rocking, among others. They manifest in different ways, depending on individuals, settings, and time conditions [3].

Different technological tools with various shapes and features have been employed in supporting sessions with children with ASD. Indeed, the use of technological devices was already proven to provide a stimulus for children belonging to this spectrum, promoting social interaction [4,5]. Furthermore, in general, most of the works are focused on exploring the interaction between children and robots on tasks such as imitation and collaborative interaction [6,7]. Conversely, in general, these studies use non-humanoid robots or systems with no (or at least low) ability to adapt to the activity. In this sense, some of the interactions with robots may be rigid, ambiguous, and confusing since most of the systems are teleoperated or controlled via the Wizard of Oz method [8,9,10]. Additionally, these approaches can impose an additional cognitive load on the researcher/therapist during the supporting sessions.

Conversely, successful human-human communication depends on the ability of both partners to read affective and emotional signals. Therefore, in order to enrich the interaction between a robot and a user, it is paramount for the robot to receive some form of feedback from its peer actions in order to better adapt its behavior to the user. Affective computing tries to create a link between the emotionally expressive human and the emotionally lacking computer [11]. It can be used to understand emotional communication in typically developing children and children with ASD [12,13,14]. Furthermore, it allows the introduction of some adaptation to these platforms, enriching the interaction with the user and reducing the cognitive burden on the human operator. A supervised behavioral system architecture using a hybrid approach to allow the detection of the child’s behavior was proposed in [15] by the research team. This method has the main goal of enabling a more natural interaction by adapting the robot’s behavior to the child’s action. This framework takes into account children’s movements, such as typical or stereotypical behaviors, in order to determine, for example, the child’s interest in the activity.

Following this idea, the present work proposes a method to automatically detect in real-time typical and stereotypical actions of children with ASD. The system uses the Intel RealSense and the Nuitrack SDK to detect and extract the user joints coordinates. Then, a Convolutional Neural Network (CNN) learning model trained with different behavior actions is used to classify the different patterns of behavior. The principal novelty lies in applying this methodology to stereotypical behaviors of the ASD target group. A dataset, Typical and Stereotypical Behaviours Dataset (TSBD), containing typical and stereotypical behaviors was developed with 32 children. Furthermore, the recognition pipeline operates on 3D sensor data in real-time, being lightweight enough to run on lower resources hardware. Additionally, some of the current approaches that use CNN first convert the joints data that are obtained from a 3D sensor into an image representation, i.e., the spatial and temporal information are mapped into a 2D image that then is used as input to a CNN model. The order of the joints data in the final image usually follows the human body configuration, meaning that the joints are grouped together in limbs, and one trunk as the human body is composed of four limbs and one trunk, for example, the right shoulder, elbow, and hand are grouped into the right arm limb [16,17,18,19]. Therefore, as an additional novel contribution, the present work analyzed if the joints’ sequence could influence the model’s performance. To verify this, two experiments were conducted, and statistical methods were used in the analysis.

This paper is organized as follows: Section 2 presents the related work; Section 3 shows the proposed approach; the experimental results are presented and discussed in Section 4; the conclusions and future work are addressed in Section 5.

## 2. Related Work

Human Action Recognition (HAR) is a broad key field in computer vision. It is employed in video surveillance, human-machine interactions, and robot vision. There are numerous research directions in HAR. Traditionally, studies in HAR have mainly focused on the use of hand-crafted features [20,21] that can be provided by 2D cameras (RGB data) or 3D sensors (RGB + Depth information). Works with RGB data only, usually start by foreground extraction to detect the action region, to then extract features, by employing techniques such as Cuboids [22] or HOG/HOF [21]. Probabilistic graphical models such as Hidden Markov Models (HMM) or the Dynamic Time Warping (DTW) algorithm, which measures the similarity between two temporal sequences, are used to classify the action [22,23]. Afsar et al. [22] proposed a method to automatically detect a subject and perform action recognition using a Hidden Markov Model and a bag of Words from RGB images from surveillance cameras. The Gaussian mixture model was used to perform background subtraction. The model achieved an accuracy of 97.02% on their dataset with two classes (‘Sit’ and ‘Walk’). The authors in [24] applied a novel method for action recognition using trajectory-based feature representation, tracking spatial-temporal interest points (STIPs) using a Cuboid detector to then build trajectories using SIFT-matching between STIPs in consecutive frames. A Support Vector Machine (SVM) was used to classify the actions. The method was tested on the UCF sports dataset [25], achieving an accuracy of 89.97%. Nonetheless, one of the major drawbacks of the 2D data is the absence of 3D structure from the scene [16]. Furthermore, by only using the RGB information, these approaches tend to be more influenced by the environment lighting conditions.

With the rapid development of 3D sensing technology, several works propose the use of this hardware for different approaches [26] in order to tackle the HAR problem. Furthermore, devices such as Microsoft Kinect [27] and Orbec Astra [28] offer, through their SDKs, real-time skeleton detection, and tracking algorithms, an effective way to accurately describe actions. The main advantage of skeleton-based representations is the lower dimensionality of the data in comparison with representations based on RGB/RGB-D, allowing the action recognition systems to be computationally faster and less complex. Thus, several skeleton-based action recognition methods have been proposed [29,30], being a promising research direction. The authors in [31] proposed an approach that consisted of computing hand-crafted features by combining 3D joints moving trend and geometry. These features were used as input to a Linear SVM classifier. In that case, the method achieved an overall accuracy of 91.3% on a public dataset. Following this trend, some works in the literature have been using skeleton-based methods in order to detect some stereotypical behaviors in children with ASD [32]. The authors in [32] used the Microsoft Kinect V1 and the DTW algorithm to detect the hand flapping or hand wave gesture, achieving an overall test accuracy of 51.0%. However, these approaches suffer from a lack of automation because of the dependency on hand-crafted features, requiring pre-processing the input data.

More recently, approaches based on Deep Learning techniques have been used and have yielded remarkable results in many recognition tasks. Most popular approaches use Recurrent Neural Networks with Long Short-Term Memory units (RNN-LSTM) for skeleton-based action recognition [33,34,35], achieving high-level performance and showing superior results when compared to classical hand-crafted approaches. However, they tend to overemphasize the temporal information and lose the spatial information of skeletons [36]. Additionally, the amount of training time is considerably more when training LSTMs than CNN models, mainly because GPUs are optimized to process 2D data with extreme parallelism and speed, which CNNs utilize. Bai et al. [37] show that a CNN could achieve similar or even better performance than an RNN in many tasks such as speech recognition [38], some tasks of NLP [39], among others. Following this trend, recent works in the literature have been employing Convolutional Neural Networks (CNNs) to tackle the HAR problem [40]. Many studies also indicate that CNNs are better than hand-crafted approaches since they have the ability to learn complex motion features [33,41,42]. However, most existing CNN-based methods use only the RGB component, only the depth component, or a combination of both components (RGB-D) as the input to learning models. Indeed, RGB-D images are informative for action recognition, but due to the large dimension of the input features, their use increases the computation complexity of these models rapidly. Additionally, the computational burden can be increased by adding more layers, partially affecting the real-time response during the test and training phases, especially when fine-tuning the CNN models. Thus, resulting in more complex and slower models, less practical for solving large-scale real-time problems. In order to tackle the dimensionality problem and computation complexity, works in [16,17,36,43] use a skeleton-based representation with deep learning techniques for the recognition of different behaviors. The work proposed by [17] consists of using skeleton-based representation data obtained from a 2D camera by using OpenPose [44] for skeleton detection in order to recognize three main actions: Idle, Wave, and Walking. The pipeline consists of first detecting the human by using a YOLO V3 [45] followed by skeleton detection and tracking to finally recognizing the action. The method achieved an overall accuracy of 97.1% in their use case, recognition of three actions in a parking lot scenario to be employed in automated vehicles. Although it is a step forward to reduce the model complexity and running time, this approach requires dedicated hardware (a dedicated GPU to run the models was used). Thus, future improvements in the pipeline configuration are necessary to optimize system performance. Another approach [16] consisted of using a 3D sensor, the Microsoft Kinect V2, to extract the user joints coordinates. Then, the 3D data was converted into image space. This method speeds up the overall pipeline since the Kinect SDK detects and extracts in real-time the user joints coordinates. The authors used different configurations of the ResNet architecture by varying the deepness of the model. The architecture was evaluated on a dedicated NVIDIA GTX 1080 Ti GPU achieving an overall accuracy of 99.9% and 99.8% on the MSR action 3D [46] and KARD [47] datasets, respectively. Although the accuracy is high and the classification time for a skeleton sequence is around 0.128 s, it requires the use of a dedicated GPU with a final model configuration of 44 layers. The solutions proposed in [16,17,36,43] used dedicated hardware, having a considerable computational burden in both training and testing phases, compromising real-time performance.

Table 1 summarizes some recent approaches that employ skeleton-driven action classification.

## 3. Methodology

One of the goals of this work is to enrich the interaction between children with ASD and a robotic platform by recognizing user behavior intentions and adapting the robot behavior accordingly. One way to recognize different actions of the user is through skeleton pose data from depth sensors. To recognize in real-time the typical and stereotypical behaviors of children with ASD, it is important to take into account the hardware to be used as well as to select the behaviors to be detected. Thus, it was also analyzed if the sequence of the joints may influence classifier accuracy. This section presents the proposed system and the pipeline, as well as the construction of the dataset used.

### 3.1. Proposed System

The presented approach to be used in the solution was proposed by the authors in [15]. Thus, the hardware was selected, taking into account the portability of the final system and the real-time requirements. Following this idea, the proposed system consists of an Intel RealSense 3D sensor [53] and a computer. The Intel RealSense depth camera used in the present work is the D435, which enables capturing stereo depth up to 90 frames per second (fps), with a wide field of view (depth diagonal FOV > 90°), by using the active IR stereo depth technology. The camera includes a dedicated vision processor that allows for computing stereo depth data in real-time. It has a 1080p RGB camera, global shutter technology, and long-range capabilities (up to 10 m). The D435 version was mainly chosen because of its small size (90 mm × 25 mm × 25 mm) in comparison to other 3D sensors. It is easily portable and connects to a computer with a single cable (offering power and data) over USB 3.0 Type-C. 

**Figure 1:**
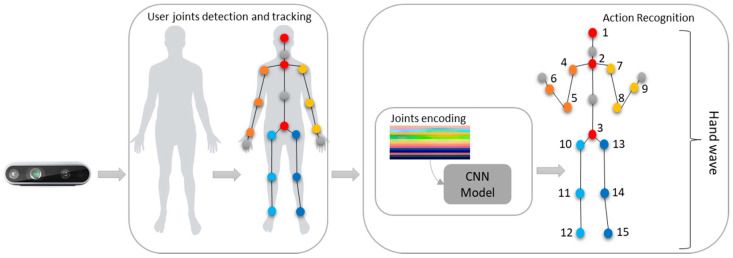
The overall system pipeline. It starts by detecting and tracking the user joints through the Nuitrack SDK. Then, the data is served as an input to the learning model to classify the action. The joints used are highlighted in orange (left arm, shoulder, elbow, and wrist joints), yellow (right arm, shoulder, elbow, and wrist joints), light blue (left leg hip, knee, and ankle joints), dark blue (right leg hip, knee, and ankle joints), and red (head, neck, and spine joints).

In order to track the user and detect the joints in real-time, it was used the Nuitrack SDK [54]. Nuitrack is a 3D tracking cross-platform middleware capable of full-body skeletal tracking (up to 19 joints) (Figure 1), with an agnostic 3D sensor independent by supporting the Microsoft Kinect V1 and V2, Orbbec Astra, Intel RealSense, among other 3D devices. The solution can track up to 6 skeletons at a time.

The hardware used consisted of a notebook equipped with an Intel(R) Core (TM) i7-8650 quad-core CPU with 16 GB of RAM.

### 3.2. Pipeline and Processing

The overall system pipeline is presented in Figure 1. It starts by detecting the user and tracking the body joints. This process is done by using the Nuitrack SDK and the data from the Intel RealSense depth device. From the 19 joints that are returned by the SDK, 15 are selected: the right and left arm shoulder, elbow and wrist joints, the right and left leg hip, knee, and ankle joints, and the head, neck, and spine joints. The hand joints were excluded due to some inaccuracy when tracking them.

The selected joints are highlighted in orange (4, 5, and 6), yellow (7, 8, and 9), light and dark blue (10, 11, and 12 and 13, 14, and 15), and red (1, 2, and 3) in Figure 1. Finally, the joints data obtained over several frames were used as input for the action recognition model to classify the action.

To explore skeleton-based action recognition with CNN methods, it is necessary to represent a temporal skeleton sequence in an effective way to then feed it into a CNN model. Thus, the idea relies on encoding the spatial and temporal dynamics of skeleton sequences onto a 2D image structure. By using the static postures and their temporal dynamics, they can be shaped into a static color image structure [16]. This color image representation can be fed into a CNN, enabling it to learn image features and classify them into classes to recognize the original sequence of skeleton data. 

**Figure 2:**
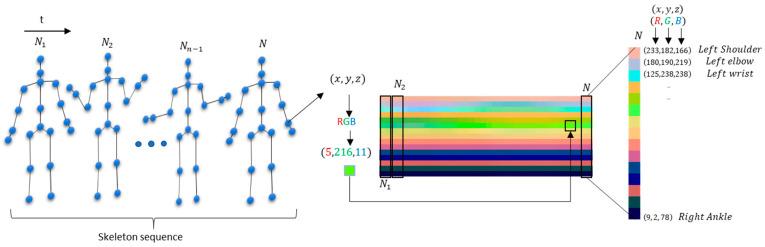
The joints are encoded by color in the image space, where N is the number of frames. For example, the encoding process for the wrist joint is highlighted in the middle, where the *x*, *y*, and *z* coordinates are normalized by using Equation (1) into the range of 0–255. Additionally, on the right, the encoding process for the N skeleton frame is highlighted in more detail. The final image dimensions are 15 joints (vertical axis) × 32 frames (horizontal axis) × 3 channels (comprising the converted coordinates to RGB).

The encoding process of a skeleton sequence (S) with N frames into an image space representation takes place in this way: each 3D joint coordinate (*x*, *y*, and *z*) available for a skeleton sequence in a given frame (*f*) is normalized into the range of 0–255, $k{\left(f\right)}^{\prime },$ by applying Equation (1):

(1) $$k\left(f\right)\prime =255\times \frac{k\left(f\right)-\min\left\{c\right\}}{\max\left\{c\right\}-\min\left\{c\right\}}$$

where *k* is the coordinate (*x*, *y*, or *z*) to be normalized, and min{*c*} and max{*c*} are the minimum and maximum values of all coordinates in the sequence, respectively. The encoding process can be seen in Figure 2. In this new image representation, the three components (R, G, B) of a color pixel are the transformed skeleton joints coordinates (*x*, *y*, *z*)*—x* = R; *y* = G; *z* = B, i.e., the raw skeleton data of sequences are converted in 3D tensors, which is used as the input features for the learning model.

All skeleton sequences in image space has a 32 × 15 × 3 size, where 32 is the number of frames and the width of the image, 15 is the number of joints representing the height of the image, and 3 is the number of channels. The sequence size of 32 frames was chosen to analyze one to two seconds of movements to estimate an action.

**Figure 3:**
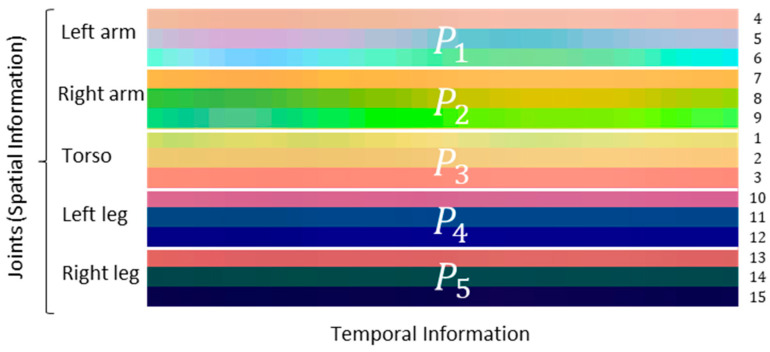
Representation of the pixels in image space according to the human body’s physical structure. Each colored strip represents the spatial position of a numbered joint (on the right) over the frames.

The human body is composed of four limbs and one trunk. This defines how actions are performed—a simpler action can use one limb, where more complex actions involve the coordination of two or more limbs and the torso. Following what is proposed in [16], each skeleton frame is divided into five groups—two arms ($P_{1}$, left arm, and $P_{2}$, right arm), torso ($P_{3}$), and two legs ($P_{4}$, left leg, and $P_{5}$, right leg)—which allows to keep the local motion characteristics, generating more discriminative features in image-based representations (Figure 3).

**Figure 4:**
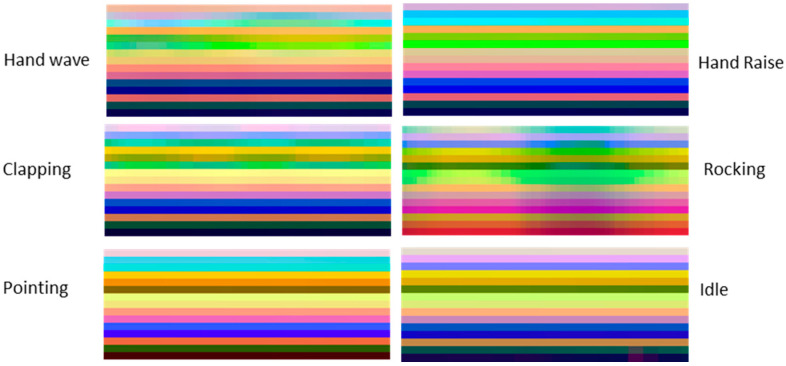
Some samples of skeleton-based image representations from the TSBD dataset used in this work.

Figure 4 shows samples of skeleton-based representations of some actions from the TSBD dataset, detailed in Section 3.3.2.

### 3.3. Datasets

In the present work, two datasets were used: the KARD dataset [47] and the TSBD dataset developed with typical and stereotypical behaviors based on the previous works of the research team with children with ASD [9].

#### 3.3.1. KARD Dataset

The KARD (Kinect Activity Recognition Dataset) [47] contains 18 activities, where each activity is performed 3 times by 10 different subjects. The activities are horizontal arm wave, high arm wave, two hand wave, catch cap, high throw, draw x, draw tick, toss paper, forward kick, side kick, take umbrella, bend, hand clap, walk, phone call, drink, sit down, and stand up. The skeleton joints used were head, neck, torso, right shoulder, right elbow, right hand, left shoulder, left elbow, left hand, right hip, right knee, right foot, left hip, left knee, and left foot.

#### 3.3.2. TSBD Dataset

The Typical and Stereotypical Behaviours Dataset (TSBD) used includes typical and stereotypical behaviors that children with ASD usually present. The stereotypical actions are usually manifested when they are exposed to new environments or when there is a change in their daily routine and, as a result, they have a tendency to block the excessive feelings by exhibiting stereotyped behaviors, as hand-flapping (or hand wave), rocking, covering the ears, among others [2]. Based on a previous work of the research group [9] with 15 children interacting with a humanoid robot, three stereotyped behaviors (hand wave, covering the ears, and rocking) were selected as the basis to develop the TSBD dataset, together with other 6 typical actions—idle, standing, clap, hand raise, pointing, and turn. These behaviors are also usually expressed by children with ASD during activity [9].

**Figure 5:**
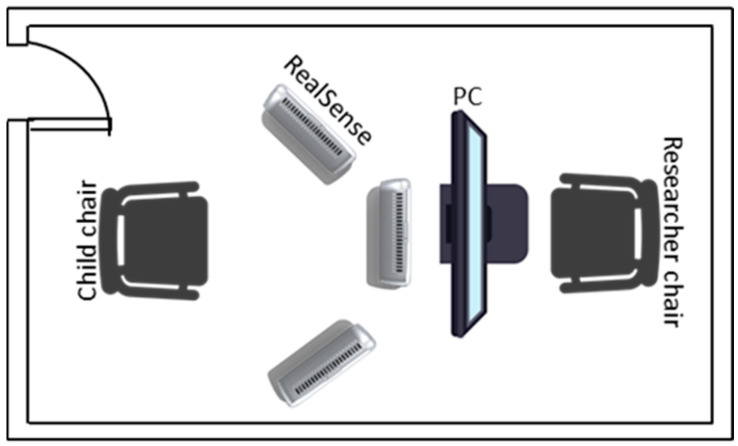
The experimental configuration used to extract the data for the dataset. The data was extracted by placing the Intel RealSense in three configurations: in front of the children, on his/her left, and his/her right.

An experimental setup was developed to collect the data. The Intel RealSense device was placed in three different configurations, as seen in Figure 5.

The participants considered for the dataset construction were 32 typically developing children aged between 6 and 9 years old. Each child was seated in front of the Intel RealSense device and, for each action, performed 3 frontal samples and two side samples (from the left and right side). A total of 1440 samples were collected (160 samples per class).

### 3.4. Deep Learning Network Architecture

**Figure 6:**
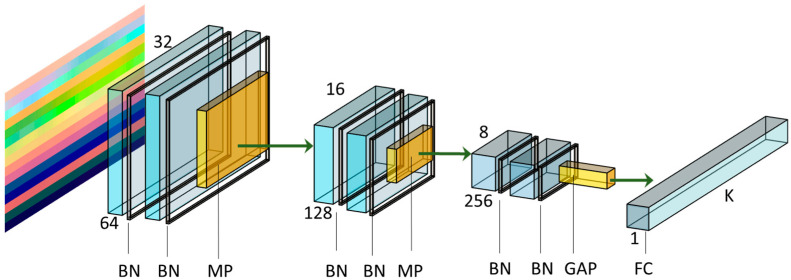
Network architecture used in the present work. BN: Batch Normalization; MP: Max Pooling; GAP: Global Averaging Pooling; FC: Fully Connected layer. The values 64, 128, and 256 are the number of filters used in each convolutional layer. The values 32, 16, and 8 are the resulted tensor width after the MP layer. As an example, the image representation (on the left) serves as input to the model. Adapted from [17].

The DL network architecture chosen, depicted in Figure 6, consisted of six convolutional layers, each with a 3 × 3 kernel, as well as padding *p* = 1 and stride S = 1. The architecture used was based on the work proposed by [17]. Although a large spatial filter (such as 5 × 5 or 7 × 7) can provide an advantage in terms of their expressiveness and ability to extract features at a larger scale, it comes with a high computation cost. It has been shown [55] that a 5 × 5 convolution can be more computationally efficient, represented by two stacked 3 × 3 filters. Therefore, the network architecture implemented has three times two stacked convolutional layers in order to extract features at a larger scale, followed by a batch normalization layer [56], and the rectified linear unit (ReLU) was used as the activation function in the convolutional layers. A max-pooling layer with kernel size 2 × 2 was used after the second and fourth convolutional to reduce the spatial resolution by a factor of two. Then, after the last convolutional layer, a Global Average Pooling (GAP) layer was used. Since Fully Connected (FC) layers are susceptible to overfitting, which can compromise the generalization ability of the network, it was suggested to use global averaging of pooled layers to counteract this effect [57]. They have the advantage of having no parameter to optimize; thus, overfitting is avoided at this layer. Moreover, global average pooling sums out the spatial information. So, it is more robust regarding spatial translations of the input.

Finally, an FC layer was placed at the end to perform the final action classification. By being a small architecture, the training was fast due to few parameters with a short inference time, resulting in an overall small model while maintaining high accuracy. The network was trained on KERAS using a TensorFlow backend.

## 4. Experimental Results and Discussion

To infer if the joints sequence might impact the classifier performance, several skeleton configurations were evaluated based on the classifier accuracy achieved for the two datasets (KARD dataset and the TSBD dataset developed with typical and stereotypical actions).

Two main experiments were conducted:

- E1 compares the accuracy of the skeleton configuration $B_{1}$ (control group) with other five skeleton combinations ($B_{2}$, $B_{3}$, $B_{4}$, $B_{5}$, and *B*_6_), where the sequence of the group (*P*_1_ to *P*_5_) was changed;
- E2 considered the joints of four skeleton combinations ($C_{2}$, $C_{3}$, $C_{4}$, and $C_{5}$), each with 15 joints randomly sequenced and again compared with the skeleton configuration $B_{1}$.

The joints were grouped according to the representation displayed in Table 2, where the groups are shown with the numbered joints following the same configuration presented in Figure 1 and Figure 3. For E1, the skeleton configurations used are shown in Table 3. For E2, the sequence of the joints was randomly obtained for each skeleton configuration as shown in Table 4. The goal was to see if the sequence of the joints in the image representation impacted the model performance.

In order to verify if the skeleton configuration could impact the accuracy of the model, a statistical analysis was performed based on the ANOVA (F-statistics). Then a pair-wise analysis was conducted using independent Student’s *t*-test (t-statistics), when necessary, to understand the direction of the difference between the control group ($B_{1}$) and the other skeleton configurations.

The results concerning the classifier performance are also addressed. Cross-subject evaluation was performed in both stages of the present work.

The model was trained for 200 epochs, with a batch size of 64 and a learning rate of 0.05. A weight decay (L2 regularization) of 1 × 10^−4^ was used to counteract overfitting. Stochastic Gradient Descent (SGD) was used as an optimizer. The learning rate starts at 0.01, and it was reduced by a factor of ten every 50 epochs. The network was trained over 20 runs to ensure the reproducibility of the results and exclude random effects due to the training process (random initialization of layer weights, random shuffling of data, and batch creation). Therefore, results are reported in terms of mean value with standard deviation (mean value ± s.d.%).

### 4.1. KARD Results

**Figure 7:**
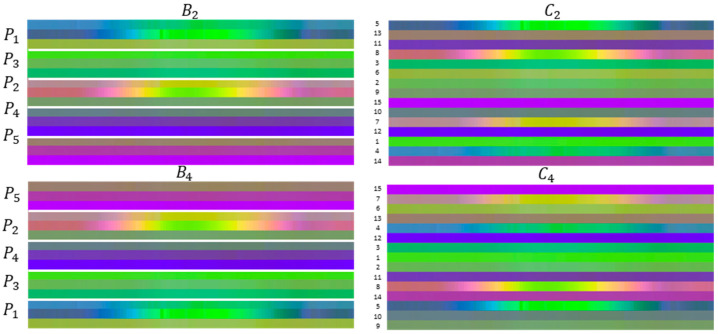
Some samples of skeleton-based image representations from the KARD dataset. On the left side, it is shown the images for the skeleton configurations $B_{2}$ and $B_{4}$. On the right side, the images for the skeleton configurations $C_{2}$ and $C_{4}$ are displayed.

The KARD dataset was randomly divided into two datasets: the data of seven subjects were selected for training and three subjects for the validation data. The experiments E1 and E2 were conducted. Figure 7 shows the skeleton-based image representations for $B_{2}$, $B_{4}$, $C_{2}$, and $C_{4}$ for the class ‘two-hand wave’.

For all skeleton configurations evaluated in E1, the differences are statistically significant in terms of accuracy (F(5) = 418.41, *p* < 0.001), where $B_{1}$ had the highest accuracy (94.0% ± 0.4%) (based on a pair-wise evaluation between $B_{1}$ and the other five skeleton configurations). Additionally, for skeleton configurations evaluated in E2 a similar conclusion was obtained (F(4) = 432.67, *p* < 0.001), where $B_{1}$ presented the highest accuracy (94.0% ± 0.4%).

**Figure 8:**
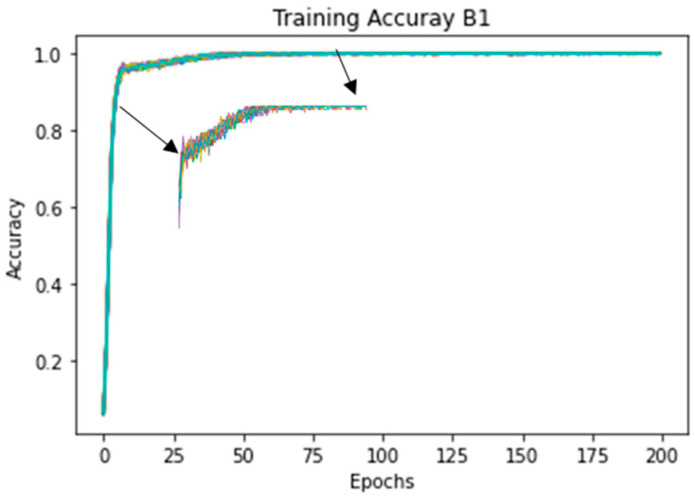
Plot of the training accuracy over the 20 runs. The accuracy for the first epochs (5 to 100) is highlighted.

**Figure 9:**
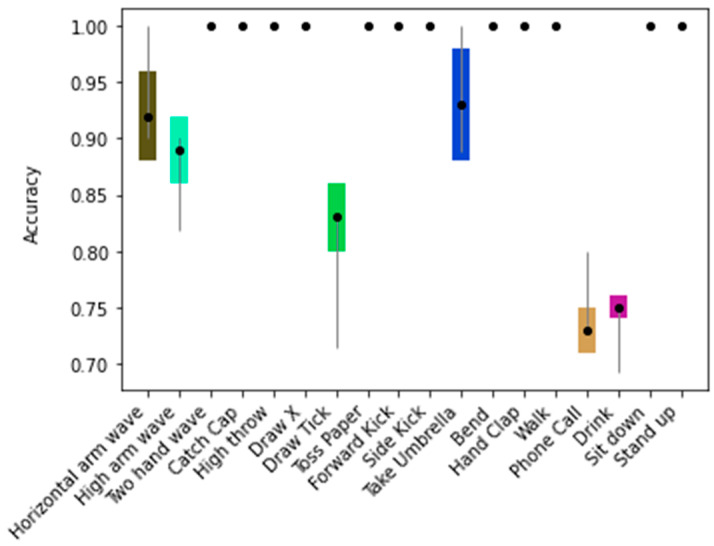
The error plot bar of the mean accuracy per class as well as the standard deviation for the model trained with the skeleton configuration $B_{1}$.

Since the skeleton configuration $B_{1}$ presented the highest mean accuracy (94.0% ± 0.4%), Figure 8 shows the training accuracy over the 20 runs, and Figure 9 shows the error plot bar of the mean accuracy per class as well as the standard deviation for each of the 18 classes over 20 runs.

In general, the classes achieved mean accuracies of 100.0% (Figure 9) on the test data. Only 6 out of the 18 classes achieved a lower mean accuracy, namely: Horizontal arm wave (92.0% ± 4.0%), High arm wave (88.3% ± 3.2%), Draw tick (82.1% ± 3.6%), Take umbrella (92.8% ± 5.3%), Phone call (73.8% ± 2.6%), and Drink (74.1% ± 2.1%).

The Matthews Correlation Coefficient (MCC) was also used to assess the classifier performance. The model achieved an MCC of 93.7% ± 0.0%.

### 4.2. TSBD Dataset Results

**Figure 10:**
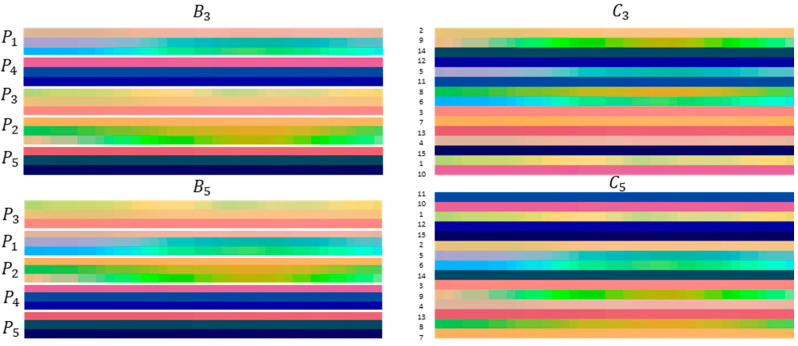
Some samples of skeleton-based image representations from the TSBD dataset. On the left side, it is shown the images for the skeleton configurations $B_{3}$ and $B_{5}$. On the right side, the images for the skeleton configurations $C_{3}$ and $C_{5}$ are displayed.

The original dataset was randomly and balanced divided into three datasets: 20 subjects were selected for training, 5 subjects were selected for validation, and 7 subjects for the test data. The experiments E1 and E2 were performed. Figure 10 shows skeleton-based image representations for $B_{3}$, $B_{5}$, $C_{3}$, and $C_{5}$ for the class ‘HAND_WAVE’. 

#### 4.2.1. Statistical Analysis

For all skeleton configurations evaluated in E1, the differences are statistically significant in terms of accuracy (F(5) = 17.34, *p* < 0.001), being the $B_{1}$ with the highest mean accuracy (93.8% ± 0.6%). However, for the pair-wise skeleton configuration $B_{1}$ and $B_{6}$ the difference is not statistically significant (t(38) = 0.38, *p* = 0.35) considering the validation dataset. Regarding the test dataset, similar results were obtained, that is, statistical differences in terms of accuracy were found (F(5) = 95.09, *p* < 0.001), with $B_{1}$ with the highest mean accuracy (92.4% ± 0.0%).

Additionally, for skeleton configurations evaluated in E2 statistical differences were found in terms of accuracy in both validation and test datasets (F(4) = 20.10 and F(4) = 107.95, respectively, *p* < 0.001), where $B_{1}$ presented the highest accuracy (93.8% ± 0.6% for the validation dataset and 92.4% ± 0.0% for the test dataset), based on a pair-wise evaluation between the $B_{1}$ and the other skeleton configurations. Indeed, the inter-class accuracy when comparing the control group ($B_{1}$) with one of the skeleton configurations that presents one of the lowest mean accuracies ($C_{2}$); in general, all classes mean accuracy drops (Table 5), and the class ‘COVER_EARS’ presents the most noticeable drop in terms of mean accuracy (from 93.2% ± 0.6% in $B_{1}$ to 83.2% ± 2.3% in $C_{2}$).

#### 4.2.2. Class Activation Maps

**Figure 11:**
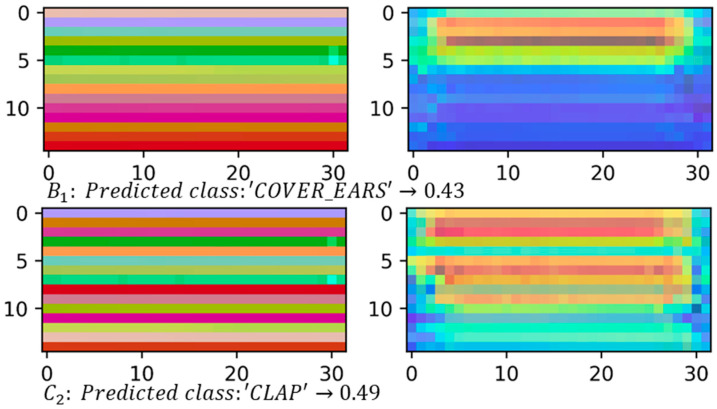
Skeleton-based image representations for the ground-truth class ‘COVER_EARS’ from the TSBD dataset. The predicted class and its score are shown below each sample on the left. On the right, it is possible to see the activation maps for each sample. It can be observed that the highlighted regions vary depending on the skeleton sequence, e.g., for $B_{1}$ the upper part of the region in the image is highlighted. On $C_{2}$ the regions highlighted are more spread.

**Figure 12:**
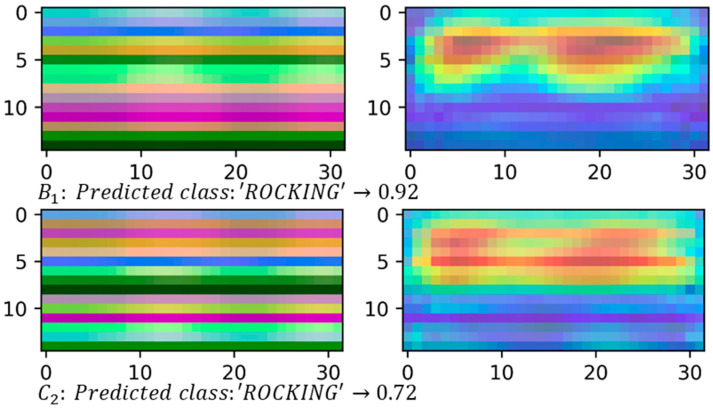
Skeleton-based image representations for the ground-truth class ‘ROCKING’ from the TSBD dataset. The predicted class and their score are shown below each sample on the left. On the right, it is possible to see the activation maps for each sample. It can be noticed that the highlighted areas are, in general, in the same region on the image for both $B_{1}$ and $C_{2}$.

Figure 11 and Figure 12, on the left, show some samples of skeleton-based image representations for the classes ‘COVER_EARS’ (Figure 11) and ‘ROCKING’ (Figure 12) following the skeleton sequences $B_{1}$ and $C_{2}$, respectively. On the right, class activation maps are shown for the models trained with the same skeleton configurations. The class activation map technique uses the Global Average Pooling (GAP) layer in CNNs to indicate, for a particular category, the discriminative image regions used by the CNN to identify that category [58]. It could be used to interpret the prediction decision made by CNN. It is possible to observe that according to the sequence of the joints, the CNN is triggered by different semantic regions of the image. The model trained with the skeleton sequence $B_{1}$ correctly predicted the ground-truth class ‘COVE_EARS’ with the top prediction score of 0.43. Conversely, the model trained with the skeleton sequence $C_{2}$ incorrectly predict the sample as ‘Clapping’ with the top score of 0.49. Considering the samples for the ‘ROCKING’ class, both models correctly predicted the ground-truth class. However, the model trained with samples with the joints sequence $C_{2}$ presents a lower prediction score of 0.72.

#### 4.2.3. Per Class Performance and Real-Time Tests

**Figure 13:**
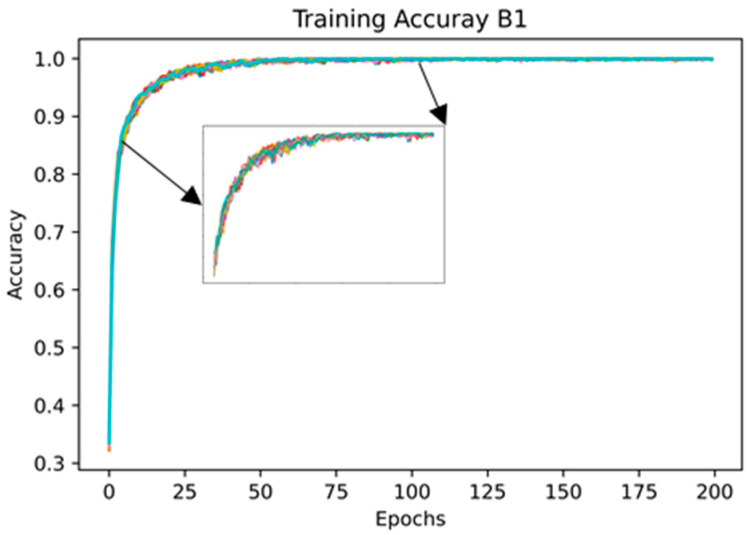
Plot of the training accuracy over the 20 runs. The accuracy for the first epochs (5 to 100) is highlighted.

Since the skeleton configuration $B_{1}$ presents the highest mean test accuracy (92.4% ± 0.0%), the following results used this configuration. Figure 13 shows the training accuracy plot of the model over the 20 runs. It is possible to see that the training converged rapidly and, in general, it remained stable over the 200 epochs.

As previously reported, the model achieved a validation accuracy of 93.8 ± 0.6% with 20% of the samples of the original dataset and test accuracy of 92.4 ± 0.0% with the test data of seven subjects.

The model achieved an MCC of 91.5% ± 0.4% on the test samples.

**Figure 14:**
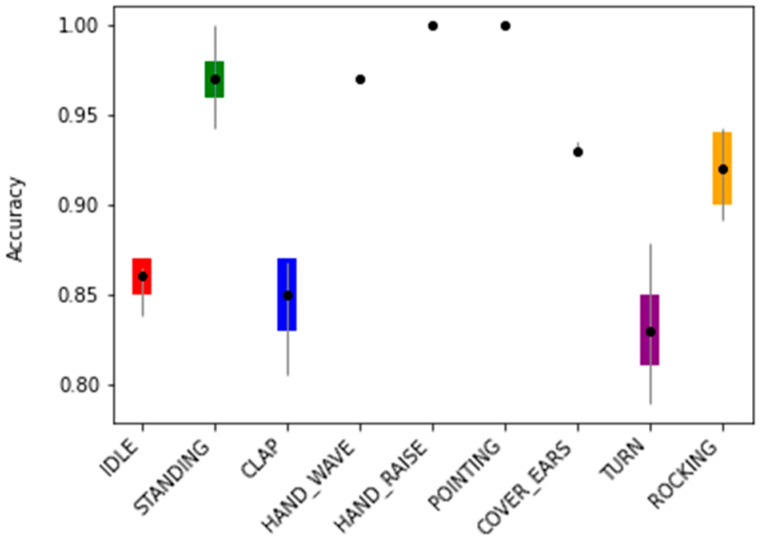
The error bar plot of the mean accuracy per class as well as the standard deviation for each of the 9 classes over the 20 runs.

Figure 14 shows the error bar plot of the mean accuracy per class as well as the standard deviation for each of the 9 classes over the 20 runs. It is possible to see that most classes had an average accuracy of over 90.0%. 

In general, the classes achieved mean accuracies over 90.0% (Figure 14 and Table 5) on the test data. The classes ‘IDLE’, ‘CLAP, and ‘TURN’ achieved, on average, the lowest accuracies (85.9% ± 1.1%, 85.1% ± 1.7%, and 83.2% ± 2.2%, respectively).

**Figure 15:**
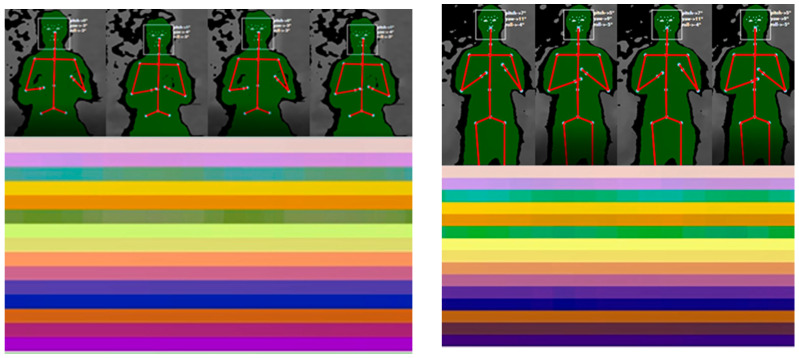
The generated image representations with the spatial and temporal information that serves as input for the model (**below**), as well as frames of the actions ‘CLAP’ (**above**) for the seated (**on the left**) and upright (**on the right**) positions. In both scenarios, the model successfully classified the action as ‘CLAP.

**Figure 16:**
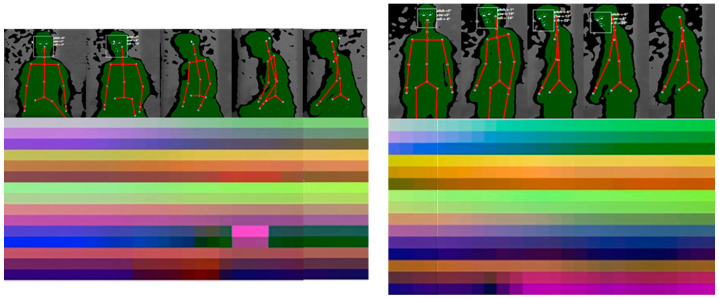
The generated image representations with the spatial and temporal information that serves as input for the model (**below**), as well as frames of the actions ‘TURN’ (**above**) for the seated (**on the left**) and upright (**on the right**) positions. In both scenarios, the model successfully classified the action as ‘TURN’.

Figure 15 and Figure 16 show the generated image representations with the spatial and temporal information that serves as input for the model, as well as frames of the actions ‘CLAP’ and ‘TURN’ performed by the subject in seated and upright positions, respectively. These representations are generated and then classified by the model in real-time. It is worth pointing out that these actions were performed by an adult subject.

### 4.3. Computational Efficiency

The computational time was calculated considering the average time for the Nuitrack SDK to retrieve the user joints coordinates plus the time to encode the joints coordinates into a sequence of frames and the classification time. The tests ran on a computer equipped with an Intel(R) Core (TM) i7-8650 quad-core CPU with 16 GB of RAM. The inference was done using OpenCV integrated with the Deep Neural Network module that allows importing saved TensorFlow models [59]. Based on the performed tests, the total maximum run time of the system is 0.032 s. Thus, the entire pipeline runs on average at about 31 FPS, which ensures the real-time capability of the system.

### 4.4. Discussion

Based on the two experiments (E1 and E2) conducted with the KARD and the TSBD datasets, it was found statistically significant differences in terms of accuracy between the control group ($B_{1}$) and the remaining skeleton configurations. Indeed, the model achieved a mean accuracy of 94.0% ± 0.4% by using the $B_{1}$ skeleton combination with the KARD dataset. Furthermore, 12 out of the 18 classes achieved a mean accuracy of 100% ± 0.0% on a benchmark dataset (as seen in Figure 9).

Based on the combinations considered, there was one exception where the differences were not found to be statistically significant for the pair-wise skeleton configuration $B_{1}$ and $B_{6}$ with the TSBD validation dataset of the present work. Concerning the test dataset, it was found statistically significant differences, with $B_{1}$ presenting the highest mean accuracy. Therefore, it was possible to infer that the sequence of the joints could impact the model’s performance. This could be further noticed when comparing the inter-class accuracy with the present work dataset between the control group ($B_{1}$) and one of the skeleton configurations with the lowest mean accuracy ($C_{2}$), where in general, all classes mean accuracy dropped. This was more pronounced in the ‘COVER_EARS’ class, where it dropped from 93.2% ± 0.6% in $B_{1}$ to 83.2% ± 2.3% in $C_{2}$.

Additionally, it is possible to observe from the activation maps (Figure 11 and Figure 12) that different regions in the images are activated by some visual pattern. For the ‘COVER_EARS’ class, this visual pattern focused more on the upper member joint position, since the upper part of the image region for the skeleton sequence $B_{1}$ was highlighted in its activation map. Conversely, for the skeleton configuration $C_{2}$ the highlighted regions were spread across the image, and the sample was incorrectly classified as ‘CLAP’. This might be due to the joints sequence being random, thus grouping the joints into limbs and trunk may allow keeping the local motion characteristics [15], particularly for some categories. Additionally, due to the nature of CNNs, it first captures local relationships, so when the joints were hierarchically ordered, the results tended to be better. This was, in general, mostly observed in the group’s joints configuration and better preserved in $B_{1}$ configuration. So, by maintaining the local and global joints hierarchy, the model ($B_{1}$) performed better in comparison to the other joint’s sequences. Considering the example for the ‘ROCKING’ class, in both cases, the predicted class was the ground-truth class, suggesting that the joints sequence did not impact as much on the model’s final prediction. However, it was possible to notice that the predicted class score was lower for the skeleton configuration $C_{2}$.

Since the skeleton configuration $B_{1}$ presented the highest mean accuracy, further tests were conducted to assess the model performance. By analyzing the remaining results, it was possible to observe that most of the classes had mean accuracies over 90% (Figure 14). However, ‘IDLE’, ‘CLAP’, and ‘TURN’ achieved mean accuracies of 85.9% ± 1.1%, 85.1% ± 1.7%, and 83.2% ± 2.2%, respectively. This may be due to some samples being similar in motion or, in the case of ‘IDLE’, some frames of the other classes could contain idle moments when the user did not move.

As shown in Figure 15 and Figure 16, the model could successfully classify the actions, considering they were executed by an adult subject from two different positions, seated and upstanding. It is worth pointing out that there was no data of adult subjects in the TSBD dataset. Furthermore, all the samples were recorded, as depicted in Figure 5, in a seated position. This could be mainly due to the normalization process that is scale and translation invariant [15]. Thus, the model was able to successfully classify the actions for these additional scenarios, effectively learning the skeleton motion characteristics.

So far, only a few works have tried to detect stereotyped behaviors of children with ASD. One of them [32] has the goal of detecting the hand wave (flapping) gesture with the Kinect sensor, achieving an accuracy of 51%. Another work [52] tried to detect other stereotypes behaviors besides the hand wave. In order to have the same basis of comparison, Table 6 compares both works with the present work in terms of accuracy for the similar studied behaviors ‘HAND_WAVE’ and ‘ROCKING’.

The present method achieved a similar performance for ‘ROCKING’, while it outperformed the other works for the detection of stereotyped behavior ‘HAND_WAVE’.

Concerning the performance of the pipeline, it runs on average at about 31 FPS on a quad-core CPU, being fast enough for most real-time applications. 

The developed model could be used with other RGBD sensors with different configurations (e.g., Kinect V1 or V2 [27], Orbec Astra [28], among others) since the Nuitrack SDK has support for these sensors [54].

## 5. Conclusions and Future Work

Individuals with ASD have several impairments concerning social communication and social interaction. Thus, it is important to propose new approaches for intervention in order to mitigate these difficulties. Furthermore, it is important for technological devices, such as robotic platforms, to receive some form of feedback from their peers’ actions to better adapt their behavior to the end-user.

Following these ideas, the present work proposes an approach to automatically detect some non-verbal behaviors. The system uses the Intel RealSense and the Nuitrack SDK to detect and extract the user joint coordinates. The proposed approach learns directly from the original skeleton data in an end-to-end manner.

The selected non-verbal behaviors for building the dataset consisted of typical and stereotypical patterns. 

In the first stage, two experiments (E1, sequence of groups, and E2, sequence of joints) with the KARD and the TSBD datasets were carried out in order to infer if the sequence of the joints in the skeleton image-based representation might impact the model accuracy. It was found statistically significant differences in terms of accuracy between the control group ($B_{1}-P_{1},\;P_{2},\;P_{3},\;P_{4},\;\mathrm{and}\;P_{5}$) and the remaining skeleton configurations. Thus, indicating that the sequence of the joints can impact the model’s performance. Since the skeleton configuration $B_{1}$ presented the highest mean accuracy on the test dataset, further tests were conducted to assess the model performance. The proposed model achieved an overall validation accuracy of 93.8% ± 0.6% (with data from five subjects) and a test accuracy of 92.4% ± 0.0% (with data from seven subjects). Additionally, the present approach delivers state-of-the-art performance compared to other methods on the detection of stereotyped behaviors (Table 6). More specifically, it achieved similar or even better performance when classifying stereotypical behaviors—‘HAND_WAVE’ and ‘ROCKING’.

The whole pipeline is able to work in real-time, running on average at 31 FPS entirely on an Intel(R) Core (TM) i7-8650 quad-core CPU in contrast to most state-of-the-art approaches that use dedicated GPU hardware when implementing deep learning approaches.

Future work will consist of using techniques to augment the TSBD dataset to improve the system accuracy. In addtition, other deep neural network architectures will be explored. Moreover, tests will be conducted with the target group in order to further assess the system performance and improve the robot behavior. Additionally, the model will be used in the system proposed by the research team in [15] in order to detect the child’s non-verbal actions during support sessions with a humanoid robot. It is intended to automatically adapt the robot’s behavior to the child’s action, providing a more adaptive support session to each child.

## Figures and Tables

**Table 1:** Some approaches of the state of the art that employs skeleton-driven action classification.

Author	Type of Camera	Hardware	Real-Time	Classification Algorithm	Nº of Actions	Application Area	Recognition Rate
Goncalves et al. 2012 [32]	Kinect V1	-----	Yes	DTW	1 stereotyped action	HCI and ASD	51.0%
Li et al. 2017 [41]	Kinect V1	----	----	CNN	60 actions [34] 27 actions [48]	HCI	76.2% 88.1%
Liu et al. 2017 [31]	Kinect V2	-------	-------		Three sets of 8 actions each	Human Computer Interaction (HCI)	91.3%
Ke et al. 2017 [49]	Kinect	----	-----	CNN + MTLN	60 actions [34] 8 actions [50] 45 actions [51]	HCI	79.6% 93.6% 93.2%
Pham et al. 2018 [16]	Kinect V2	NVIDIA GTX 1080 Ti GPU	Yes	ResNet	20 actions [46] 18 actions [47] 60 actions [34]	HCI	99.9% 99.8% 78.2%
Jazouli et al. 2019 [52]	Kinect V1	------	Yes	$P Point-Cloud Recognizer	5 stereotyped actions	HCI and ASD	94.0%
Ludl, Gulde and Curio 2019 [17]	RGB Camera	Intel i7-8700 6 core CPU NVIDIA GTX 1080 GPU	Yes	CNN	3 actions	Autonomous Driving	97.1%
Laraba et al. 2019 [18]	Kinect V2	Intel i7-7800X 2 × NVIDIA GTX 1080 Ti	----	Pre-trained architectures of CNN and RNNs	49 actions [34]	HCI	82.1%
Junwoo and Bummo 2020 [19]	RGB camera	NVIDEA JETSON XAVIER with dedicated Volta GPU	Yes (14 fps)	CNN	15 actions [34]	Human Robot Interaction (HRI)	71.0%

**Table 2:** Grouped joints in four limbs. ($P_{1}$, $P_{2}$, $P_{4}$, and $P_{5}$) and one trunk ($P_{3}$) following the convention presented in Figure 1 and Figure 3.

$P_{1}$	$P_{2}$	$P_{3}$	$P_{4}$	$P_{5}$
LA ^1^ (4, 5, and 6)	RA ^2^ (7, 8, and 9)	Torso (1, 2, and 3)	LL ^3^ (10, 11, and 12)	RL ^4^ (13, 14, and 15)

^1^ LA: Left arm; ^2^ RA: Right arm; ^3^ LL: Left leg; ^4^ RL: Right leg.

**Table 3:** Skeleton configurations of groups of joints used where $B_{1}$ is the control group. Here the order of the joint groups is changed for each skeleton configuration.

SC ^1^	Joint Groups Sequence				
$B_{1}$	$P_{1}$	$P_{2}$	$P_{3}$	$P_{4}$	$P_{5}$
$B_{2}$	$P_{1}$	$P_{3}$	$P_{2}$	$P_{4}$	$P_{5}$
$B_{3}$	$P_{1}$	$P_{4}$	$P_{3}$	$P_{2}$	$P_{5}$
$B_{4}$	$P_{5}$	$P_{2}$	$P_{4}$	$P_{3}$	$P_{1}$
$B_{5}$	$P_{3}$	$P_{1}$	$P_{2}$	$P_{4}$	$P_{5}$
$B_{6}$	$P_{5}$	$P_{4}$	$P_{3}$	$P_{2}$	$P_{1}$

^1^ Skeleton configuration.

**Table 4:** Skeleton configurations used where $B_{1}$ is the control group. Here the sequence of the joints was randomly changed for each skeleton configuration.

SC ^1^	Joint Number														
$B_{1}$	4	5	6	7	8	9	1	2	3	10	11	12	13	14	15
$C_{2}$	5	13	11	8	3	6	2	9	15	10	7	12	1	4	14
$C_{3}$	2	9	14	12	5	11	8	6	3	7	13	4	15	1	10
$C_{4}$	15	7	6	13	4	12	3	1	2	11	8	14	5	10	9
$C_{5}$	11	10	1	12	15	2	5	6	14	3	9	4	13	8	7

^1^ Skeleton configuration.

**Table 5:** Inter-class mean accuracy comparison between the control group ($B_{1}$) and $C_{2}$ with the present work test dataset.

SC ^1^	IDLE	SD ^2^	CLAP	HW ^3^	HR ^4^	PT ^5^	CE ^6^	TURN	RK ^7^
$B_{1}$	85.9% ± 1.1%	97.1% ± 1.1%	85.1% ± 1.7%	97.2% ± 0.0%	100% ± 0.0%	100% ± 0.0%	93.2% ± 0.6%	83.2% ± 2.2%	92.0% ± 2.3%
$C_{2}$	80.5% ± 1.7%	95.9% ± 1.7%	84.3% ± 0.2%	94.6% ± 0.0%	100% ± 0.0%	97.4% ± 0.6%	83.2% ± 2.3%	86.7% ± 2.2%	88.8% ± 2.1%

^1^ Skeleton Configuration; ^2^ STANDING; ^3^ HAND_WAVE; ^4^ HAND_RAISE; ^5^ POINTING; ^6^ COVER_EARS; ^7^ ROCKING.

**Table 6:** Comparison in terms of accuracy of other works in the literature that focus on the detection of stereotypical behavior.

Work	‘HAND_WAVE’	‘ROCKING’
N. Gonçalves et al. [20]	51.0%	-
M. Jazouli et al. [52]	91.5%	92.2%
Present work	97.2% ± 0.0%	92.0% ± 2.3%

## Data Availability

The KARD (Kinect Activity Recognition Dataset) was obtained from https://data.mendeley.com/datasets/k28dtm7tr6/1 (accessed on 14 January 2021).
